# Photobiocatalytic Oxyfunctionalization with High Reaction Rate using a Baeyer–Villiger Monooxygenase from *Burkholderia xenovorans* in Metabolically Engineered Cyanobacteria

**DOI:** 10.1021/acscatal.1c04555

**Published:** 2021-12-10

**Authors:** Elif Erdem, Lenny Malihan-Yap, Leen Assil-Companioni, Hanna Grimm, Giovanni Davide Barone, Carole Serveau-Avesque, Agnes Amouric, Katia Duquesne, Véronique de Berardinis, Yagut Allahverdiyeva, Véronique Alphand, Robert Kourist

**Affiliations:** † Institute of Molecular Biotechnology, Graz University of Technology, NAWI Graz, Petersgasse 14, 8010 Graz, Austria; ‡ Aix Marseille Univ, CNRS, Centrale Marseille, iSm2 UMR7313, 13397 Marseille, France; § ACIB GmbH, 8010 Graz, Austria; ∥ Génomique métabolique, Genoscope, Institut François Jacob, CEA, CNRS, Univ Evry, Université Paris-Saclay, 91057 Evry, France; ⊥ Molecular Plant Biology Unit, Department of Life Technologies, Faculty of Technology, University of Turku, Turku 20014, Finland; ¶ i3S, Instituto de Investigação em Saúde Universidade do Porto & IBMC, Instituto de Biologia Molecular e Celular, R. Alfredo Allen 208, 4200-135 Porto, Portugal; ∇ Departamento de Biologia Faculdade de Ciências, Universidade do Porto Rua do Campo Alegre, Edifício FC4, 4169-007 Porto, Portugal

**Keywords:** enzyme catalysis, photosynthesis, Baeyer−Villiger oxidation, biocatalysis, cyanobacteria

## Abstract

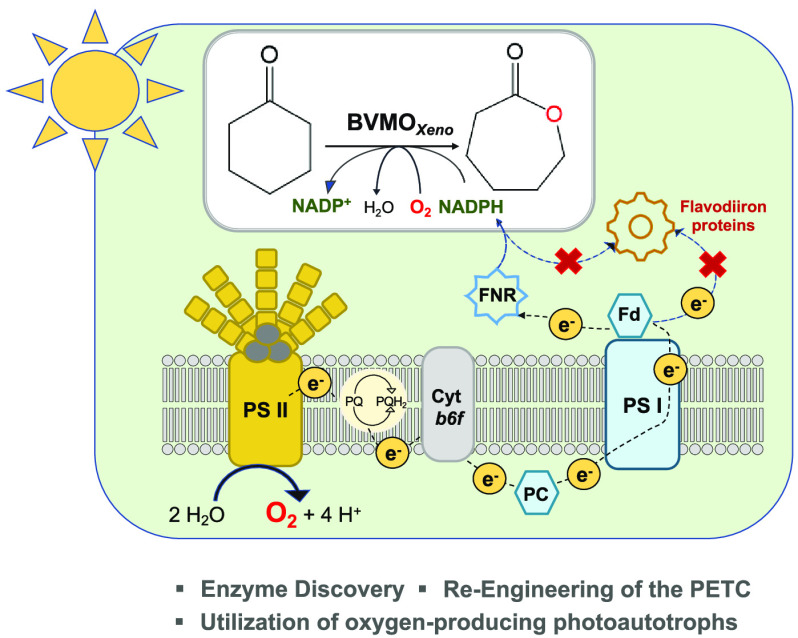

Baeyer–Villiger monooxygenases (BVMOs) catalyze the oxidation of ketones to lactones under very mild reaction conditions. This enzymatic route is hindered by the requirement of a stoichiometric supply of auxiliary substrates for cofactor recycling and difficulties with supplying the necessary oxygen. The recombinant production of BVMO in cyanobacteria allows the substitution of auxiliary organic cosubstrates with water as an electron donor and the utilization of oxygen generated by photosynthetic water splitting. Herein, we report the identification of a BVMO from *Burkholderia xenovorans* (BVMO_*Xeno*_) that exhibits higher reaction rates in comparison to currently identified BVMOs. We report a 10-fold increase in specific activity in comparison to cyclohexanone monooxygenase (CHMO_*Acineto*_) in *Synechocystis* sp. PCC 6803 (25 vs 2.3 U g_DCW_^–1^ at an optical density of OD_750_ = 10) and an initial rate of 3.7 ± 0.2 mM h^–1^. While the cells containing CHMO_*Acineto*_ showed a considerable reduction of cyclohexanone to cyclohexanol, this unwanted side reaction was almost completely suppressed for BVMO_*Xeno*_, which was attributed to the much faster lactone formation and a 10-fold lower *K*_M_ value of BVMO_*Xeno*_ toward cyclohexanone. Furthermore, the whole-cell catalyst showed outstanding stereoselectivity. These results show that, despite the self-shading of the cells, high specific activities can be obtained at elevated cell densities and even further increased through manipulation of the photosynthetic electron transport chain (PETC). The obtained rates of up to 3.7 mM h^–1^ underline the usefulness of oxygenic cyanobacteria as a chassis for enzymatic oxidation reactions. The photosynthetic oxygen evolution can contribute to alleviating the highly problematic oxygen mass-transfer limitation of oxygen-dependent enzymatic processes.

C–H oxyfunctionalization belongs to the most important class of organic transformations. With their often outstanding selectivity and capacity for the selective functionalization of hydrocarbons, oxygenases have assumed an important role in synthetic chemistry.^1^ Yet, despite their wide availability and diversity in nature, the difficulty in supplying sufficient oxygen for the reaction has, thus far, hindered the broad applications of mono- and dioxygenases. Developing enzymatic systems to establish biocatalytic processes for the production of bulk chemicals is one of the main challenges for biocatalysis; ε-caprolactone (**1b**), for example, is a precursor for the synthesis of polycaprolactone and produced at a multi-10000 ton scale per year via the UCC process.^2^ This process, which involves the oxidation of cyclohexanone using peracetic acid in stoichiometric amounts, results in a high amount of toxic side products. In contrast, Baeyer–Villiger monooxygenases (BVMO) allow for mild reaction conditions for the synthesis of various lactones and have received considerable attention as catalysts for the synthesis of important heterocyclic bulk chemicals (Scheme 1).^3,4^

**Scheme 1:**
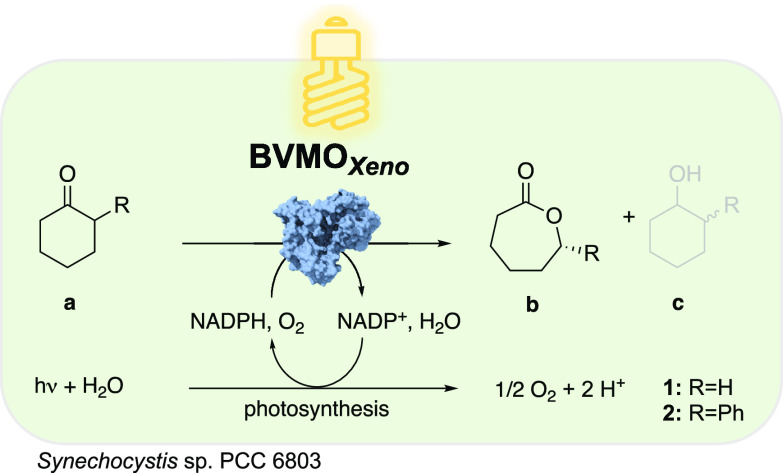
Photosynthesis-Driven Lactone Synthesis (b) and Native Alcohol Dehydrogenase Driven Ketoreduction (c) in *Synechocystis* sp. PCC 6803

The application of these enzymes on a larger scale has, thus far, been limited due to several factors; among them is the aforementioned difficulty in supplying sufficient oxygen for the reaction. Evidently, Baldwin et al. previously demonstrated that whole-cell biotransformations using BVMOs are oxygen-limited at cell concentrations as low as 2 g_DCW_ L^–1^.^5,6^

Furthermore, the stoichiometric addition of an auxiliary molecule for cofactor regeneration is necessary, which severely reduces the atom economy of the process.^7^ In a heterotrophic cell, the most common chassis for BVMOs, a considerable part of the nicotinamide cofactors are diverted toward respiration which, therefore, renders it as an additional oxygen sink. To this end, the use of photosynthetic cyanobacteria, which use photosynthetic water splitting for both oxygen evolution and the regeneration of redox cofactors, as hosts is advantageous^8^ (Scheme 1). As typical photosynthetic net oxygen evolution rates of cyanobacteria lie in the range of 1–2 μmol O_2_ mg_chl_^–1^ min^–1^, there should be sufficient oxygen available for oxyfunctionalization reactions.^8−10^

We previously reported that whole-cell biotransformations in recombinant *Synechocystis* sp. PCC 6803 (hereafter Synechocystis or Syn) expressing the cyclohexanone monooxygenase from *Acinetobacter* sp. (CHMO_*Acineto*_) under the control of a light-inducible promoter, P_*psbA2*_, exhibited reaction rates of 2–5 U g_DCW_^–1^.^11^ This represents a 10-fold lower rate in comparison to those obtained with other recombinant enzymes in the same organism.^12−15^ Moreover, the reduction of cyclic ketones by endogenous alcohol dehydrogenases (ADH) competes with the Baeyer–Villiger oxidation, which is especially problematic considering that cyclohexanol (**1c**) inhibits CHMO_*Acineto*_.^16^ Herein, we pursue an integrated approach combining enzyme discovery, promoter engineering, and redesigning of the photosynthetic electron transport chain (PETC) to increase the specific activity of BVMOs in cyanobacteria and to reduce the undesired ketoreduction.

To identify a BVMO with reaction rates in *Synechocystis* superior to that with CHMO_*Acineto*_,^11^ we expressed the genes of a panel of monooxygenases selected from a previous high-throughput cloning^17^ in *E. coli* and screened them via whole-cell oxidation of cyclohexanone (**1a**). Figure 1 shows the whole-cell biotransformation of **1a** mediated by several BVMOs expressed in *E. coli*. While 9 out of the 11 enzymes tested had a much lower specific activity in comparison to CHMO_*Acineto*_, we noted that a BVMO from *Burkholderia xenovorans* (BVMO_*Xeno*_) exhibited a higher specific rate of 19 ± 2.0 U g_DCW_^–1^ in *E. coli* (Figure 1C). This represents a ∼48% faster rate in comparison to those achieved with CHMO_*Acineto*_ (13 U g_DCW_^–1^). BVMO_*Xeno*_ belongs to the strictly NADPH dependent type I family of BVMO and has 39% identity with CHMO_*Acineto*_. Moreover, it shows the typical signature motif of type I BVMOs ([A/G]GxWxxxx[F/Y]P[G/M]-xxxD) (Figure S3).^18^

**Figure 1:**
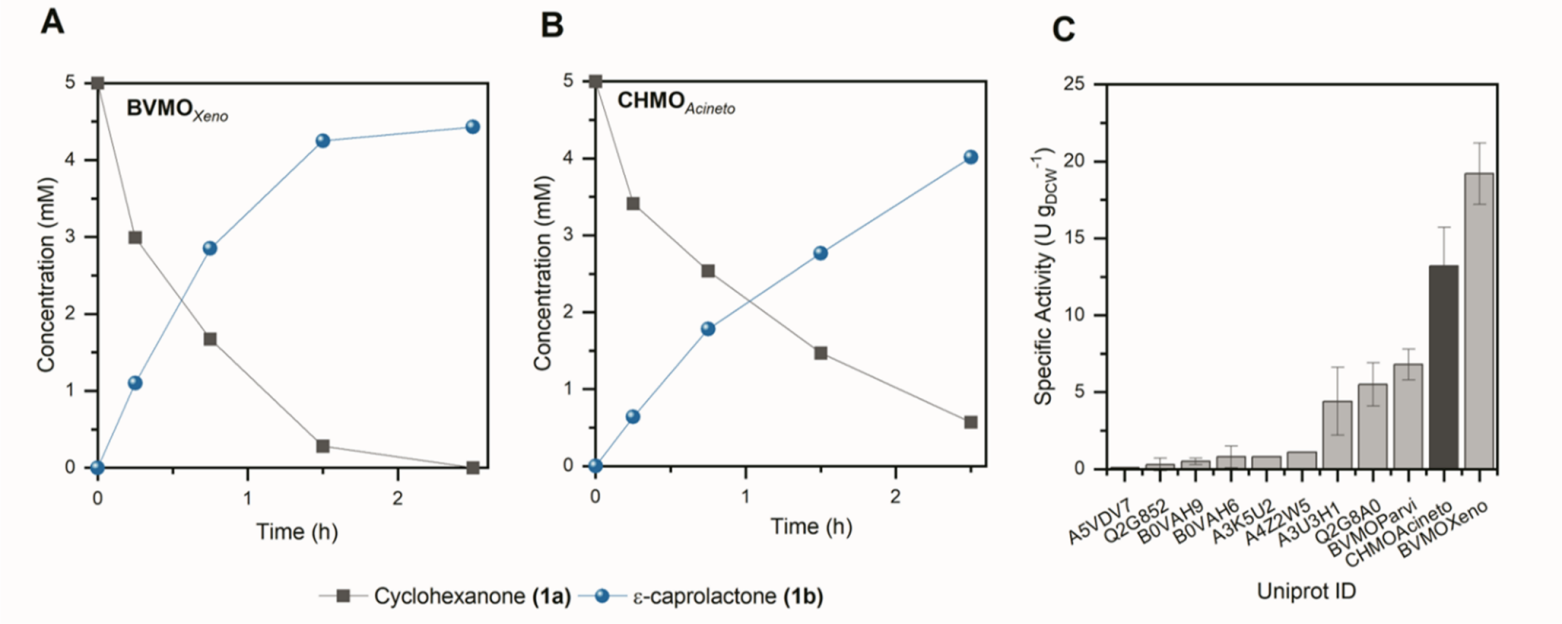
Whole-cell biotransformation of **1a** mediated by (A) BVMO_*Xeno*_ and (B) CHMO_*Acineto*_ in *E. coli* BL21(DE3). (C) Specific activities of various BVMOs in recombinant *E. coli* cells producing **1b**. Reaction conditions: 30 mL, 25 °C, 200 rpm, initial concentration of 5 mM **1a**, *N* = 3 independent repetitions. Error bars represent standard deviations.

Figure 2 shows the optimum pH and temperature of BVMO_*Xeno*_ as well as its substrate scope. The substrate scope of BVMO_*Xeno*_ is similar to that of the other type 1 BVMOs with higher activity toward cyclohexanone derivates, and the pH spectrum is typical of bacterial BVMOs. However, BVMO_*Xeno*_ has optimal activity at pH 8, while the optimum activity of CHMO_*Acineto*_ lies at pH 9.^19^ This could be an important advantage for the application in *Synechocystis* with an intracellular pH value of 7.^20^ We noted that the initial rates obtained at elevated temperatures were relatively high, with the highest rates obtained at 40 °C. This is an unusual observation for BVMOs in cell-free systems. Moreover, BVMO_*Xeno*_ showed in the ThermoFAD method^21^ in sodium phosphate buffer at pH 7.5 an unfolding temperature of 37.6 ± 0.1 °C in comparison to CHMO_*Acineto*_ with 35.6 ± 0.1 °C, which is another indication of a higher stability (Table S3). Table 1 gives a comparison of the kinetic parameters of BVMO_*Xeno*_ and CHMO_*Acineto*_ from the conversion of **1a**. We note that the catalytic efficiency of the former is higher. This is based on a 10-fold lower *K*_M_ value, whereas the specific activity is much lower. While the low stability of CHMO_*Acineto*_ in cell-free systems has been known for a long time, Rudroff and co-workers reported the surprising discovery that *E. coli* is not capable of providing sufficient cofactor for a stable maintenance of the enzyme, leading to a very poor stability also in the whole-cell system.^22^ Moreover, a visual comparison of the enzyme production in SDS-PAGE indicated a slightly better production of BVMO_*Xeno*_ (Figure S4). It remains unclear if the higher activity of BVMO_*Xeno*_ in *E. coli* cells may be attributed either to the much lower *K*_M_ value (with the efficient substrate concentration within cells being unknown) or to a higher stability of BVMO_*Xeno*_. Nevertheless, the result obtained in the whole-cell system presented this new BVMO as an ideal candidate to achieve higher reaction rates in cyanobacteria.

**Table 1:** Kinetic Parameters of BVMO_*Xeno*_ and CHMO_*Acineto*_^a^

param	BVMO_*Xeno*_	CHMO_*Acineto*_
*K*_M_ (μM)	22.7 ± 5	266.6 ± 25.5
*k*_cat_ (min^–1^)	103.0 ± 3.0	272.4 ± 5.7
*k*_cat_/*K*_M_ (μM min^–1^)	4.6 ± 0.7	1.02 ± 0.1
specific activity (μmol min^–1^ mg^–1^)	1.7 ± 0.1	8.8 ± 0.2

a Experimental conditions: 50 mM Tris-HCl, pH 8, 25 °C (see the Supporting Information for details).

**Figure 2:**
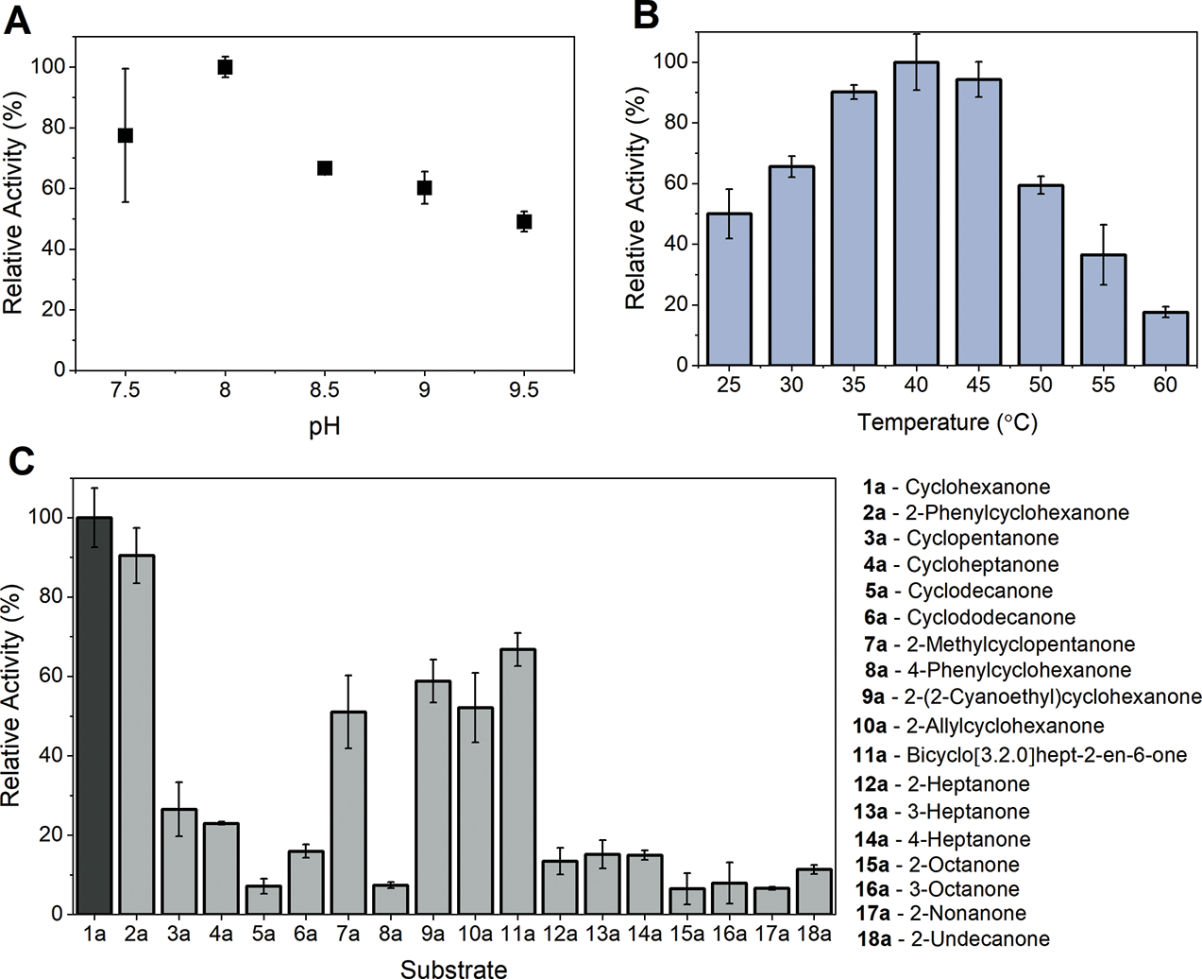
Characterization of BVMO_*Xeno*_: effect of (A) pH and (B) temperature on its activity; (C) substrate scope. Data stemmed from the purified enzyme which were measured and calculated from the NADPH consumption during the reaction with 1 mM of substrate. The relative activities were calculated in correlation with the reactions of 1 mM cyclohexanone. *N* = 3 independent repetitions. Error bars represent standard deviationa.

*Synechocystis* cells producing BVMO_*Xeno*_ under the promoter P_*psbA2*_ showed an activity of <1 U g_DCW_^–1^ (Figure S8), which is comparable to our previous results obtained with the same cells harboring CHMO_*Acineto*_.^11^ Previously, we demonstrated that the recombinant production of oxidoreductases in *Synechocystis*, such as ene-reductases^14^ and BVMOs,^11^ greatly increased reaction rates surpassing the native ketoreduction rates previously reported using wild-type cyanobacteria.^23−28^ Indeed, photobiotransformations have found application in diverse reactions, including the hydroxylation of hydrocarbons^12,15^ and synthesis of chiral amines by imine reductases, showing the versatility of the approach.^13^

While we had utilized the light-inducible promoter P_*psbA2*_ in our previous work, the stronger light-regulated promoter P_*cpc*_^29,30^ led to higher specific activities with other oxidoreductases in *Synechocystis*, thereby outcompeting the native ADHs and reducing resultant ketoreduction rates.^13,31^ This homologous promoter controls the cpc operon that encodes for a photosynthesis antenna protein, phycocyanin, and is one of the strongest promoters known for *Synechocystis*.^29,30^ Therefore, we cloned both *bvmo* genes under the control of this promoter and expressed the resulting strain under a constant light regimen. Syn::P_cpc_CHMO_*Acineto*_ showed a specific activity of 4 U g_DCW_^–1^ in the oxidation of **1a** and 0.53 U g_DCW_^–1^ in its ketoreduction. We were pleased to find that with the same promoter, Syn::P_*cpc*_BVMO_*Xeno*_, had a much higher specific activity of 18 ± 3 U g_DCW_^–1^ at a cell density of 2.4 g L^–1^ (Figure 3A,B) and an activity of 0.30 U g_DCW_^–1^ in the ketoreduction. With an initial product formation of 2.7 ± 0.4 mM h^–1^, the reaction proceeded to completion within 3 h, as shown in Figure 3A. This represented an almost 10-fold improvement in comparison to our previous work on CHMO_*Acineto*_ under the control of P_*psbA2*_, which had a specific activity of 2.3 ± 0.05 U g_DCW_^–1^ toward **1a**.^11^ Due to the lower activity in the ketoreduction, the formation of **1c** with Syn::P_*cpc*_BVMO_*Xeno*_ was greatly reduced (1.6% after 3 h) and remained constant in comparison to our previous results with Syn::P_*cpc*_CHMO_*Acineto*_. In the case of a branching metabolic pathway, where two enzymes or two groups of enzymes compete for a substrate S, the ratio of the total activities will depend (in case of low substrate saturation) on the total enzyme concentrations and the catalytic efficiencies.^32^
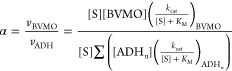
1

**Figure 3:**
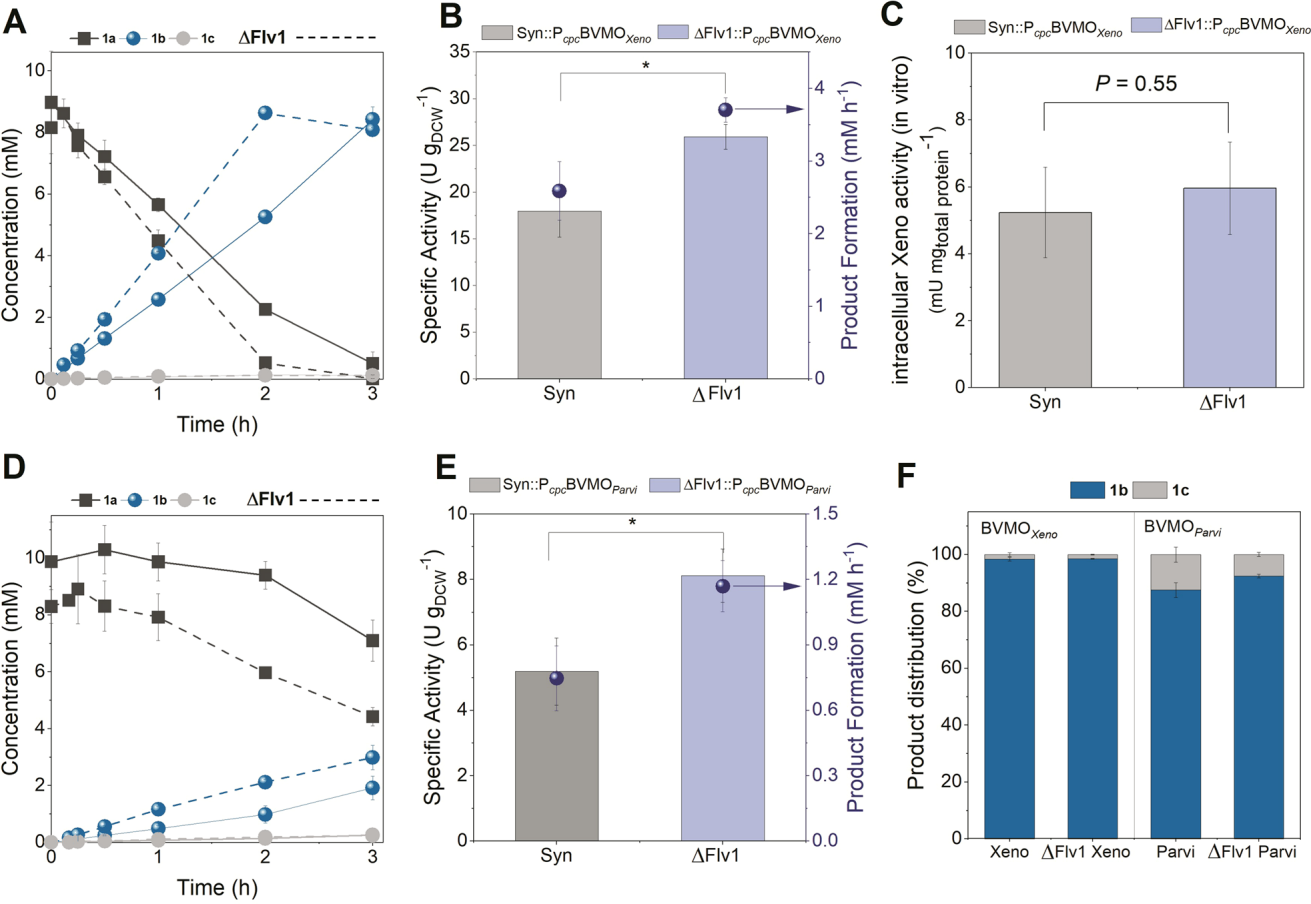
Whole-cell biotransformation of **1a** in Synechocystis harboring BVMO_*Xeno*_ and BVMO_*Parvi*_. Time course of product formation and substrate consumption by (A) Syn::P_*cpc*_BVMO_*Xeno*_ and (D) Syn::P_*cpc*_BVMO_*Parvi*_ and their corresponding ΔFlv1 mutants (depicted as dashed lines). Specific whole-cell activities relative to cell dry weight (2.4 g_DCW_ L^–1^) of *Synechocystis* harboring (B) BVMO_*Xeno*_ and (E) BVMO_*Parvi*_ and their corresponding ΔFlv1 mutants. Activity calculations were performed in the presence of ≤10% product. The rate of the product formation is depicted as a blue circle. (C) Intracellular BVMO_*Xeno*_ activity in the oxidation of **1a** using Syn::P_*cpc*_BVMO_*Xeno*_ and ΔFlv1::P_*cpc*_BVMO_*Xeno*_. (F) Product distributions after 3 h of reaction using BVMO_*Xeno*_ and BVMO_*Parvi*_. Reaction conditions: 1 mL, 30 °C, 160 rpm, initial concentration 10 mM of **1a**, light intensity of 300 μE m^–2^ s^–1^, *N* = 3 independent repetitions. Error bars represent standard deviations. *P* values were calculated using Welch’s *t* test (*P < 0.05) and correspond to the specific activity comparison between BVMO_*Xeno*_ and BVMO_*Parvi*_ and their corresponding ΔFlv1 mutants.

The Baeyer–Villiger monooxygenase and the alcohol dehydrogenase compete for the substrate. Since the dehydrogenase parameters are strain-dependent, they can be considered as constant and the *K*_M_ value of the BVMO appears as a crucial parameter for the observed selectivity. In light of this, a 10-fold lower *K*_M_ value of BVMO_*Xeno*_ toward **1a** is therefore highly important to suppress the unwanted alcohol formation.

As Syn::P_*cpc*_BVMO_*Xeno*_ showed only minimal ketoreduction, inhibition by this side product was ruled out as the rate-limiting factor for this biocatalyst. However, the reaction product **1b** might also exert an inhibitory effect. To study this, we performed whole-cell biotransformation reactions in the presence of different concentrations of **1b**. Indeed, the activity of the cells was decreased by 50% in the presence of 10 mM **1b** (Figure S9), which slows down the reaction in the later phases of the process but basically results only in a moderate extension of the reaction time.

To study the enantiospecificity of the whole-cell biocatalyst, we investigated the kinetic resolution of racemic 2-phenylcyclohexanone (**2a**), which was converted with outstanding enantiospecificity (*E* > 200, Figure S6), leading to the formation of the “normal” lactone (*R*)-7-phenyloxepan-2-one (**2b**) in very high optical purity (99% ee). Formation of the “abnormal” lactone or the undesired ketoreduction was not observed. The high selectivity demonstrates the practical value of the method for the synthesis of optically pure ketone and lactones.

In an attempt to increase the activity further, we investigated BVMO_*Xeno*_ in a *Synechocystis* mutant with a disrupted electron valve; namely, the Flv1/3 heterodimer. The regulation of photosynthetic light reactions, which produce ATP and key cofactors such as NADPH and ferredoxin, is crucial for the maintenance of redox balance within a photosynthetic organism such as *Synechocystis*. Alternative electron flow (AEF) actively partakes in this crucial process and can confer protection against various environmental stressors in the natural habitat of cyanobacteria. These routes, which alleviate excessive reduction of the photosynthetic electron and balance the intracellular ATP/NADPH ratio, may be dispensable under controlled conditions which, in turn, can broaden access to key cofactors via heterologous electron sinks.^33^ Naturally occurring flavodiiron proteins (FDPs) in *Synechocystis* act as an efficient release valve for excess electrons, reducing O_2_ to H_2_O. Although the Flv1 and Flv3 proteins have previously been shown to be crucial for survival under fluctuating light conditions,^34^ we have previously shown that, through their disruption, the activity of an NADPH-limited heterologous ene-reductase could be substantially improved at cell densities where light fluctuations are expected.^31^ Herein, we sought to expand the realm of possible reactions that can be enhanced using this approach to include oxyfunctionalizations by BVMOs.

Indeed, whole-cell biotransformations conducted in ΔFlv1 background strains had a 1.4-fold increased activity of 25.7 ± 1.2 U g_DCW_^–1^. With a product formation rate of 3.7 ± 0.2 mM h^–1^, the reaction proceeded to completion in less than 3 h (Figure 3A). Notably, cell-free extracts of Syn::P_*cpc*_BVMO_*Xeno*_ and ΔFlv1::P_*cpc*_BVMO_*Xeno*_ did not show significantly different rates in the Baeyer–Villiger oxidation of **1a**, confirming that the observed effect is not due to a higher production of the enzyme but is indeed a result of the Flv1 deletion (Figure 3C).

In order to test the robustness of the improved ΔFlv1 activities, we expressed the gene of a second BVMO from *Parvibaculum lavamentivorans* DSM 1302332 (BVMO_*Parvi*_) and tested its activity during whole-cell biotransformations. Figure 3D shows the whole-cell biotransformation of **1a** mediated by Syn::P_*cpc*_BVMO_*Parvi*_ and its ΔFlv1 variant, ΔFlv1::P_*cpc*_BVMO_*Parvi*_. Similar to results obtained with ΔFlv1::P_*cpc*_BVMO_*Xeno*_, a 1.6-fold increase in activity was observed with ΔFlv1::P_*cpc*_BVMO_*Parvi*_ (Figure 3E). BVMO_*Parvi*_, which showed an activity in the range of other BVMOs (Figure 1C), served as a good example that rational re-engineering of the PETC can improve the activities of slower BVMOs as well. Interestingly, the rate increase also reduced the formation of **1c** from 12.5% to 7.5%. In the case of the faster reaction with BVMO_*Xeno*_, the use of the ΔFlv1 mutant did not lead to any further decrease of the already low formation of 1.5% **1c** after 3 h (Figure 3F).

In conclusion, our results underline the extent to which careful selection of a candidate BVMO can help to improve reaction rates and highlight the potential of photosynthetic cofactor regeneration for enzymatic oxyfunctionalization reactions. A possible reason for the better whole-cell activity is the higher stability of BVMO_*Xeno*_ in comparison to CHMO_*Acineto*_, indicated by a higher degradation temperature and activity at higher temperatures. Furthermore, the optimal pH of 8 is closer to the intracellular pH value in comparison to the optimal pH of CHMO_*Acineto*_ at pH 9, which presumably has consequences for the functional stability of the enzyme. Finally, the much lower *K*_M_ value of BVMO_*Xeno*_ is important to achieve a higher selectivity for the Baeyer–Villiger oxidation over the ketoreduction and to almost completely suppress this unwanted side reaction.

Overall, the specific activities we obtained underscore the fact that oxygen-producing photoautotrophs can compete with oxygen-consuming heterotrophs as host organisms for biotransformations. Additionally, the rational engineering of the PETC in these organisms can serve to improve overall activities that exceed those obtained with *E. coli* at a cell density where the oxygen supply becomes limiting for this organism. After the successful rate increase by enzyme discovery, promoter, and metabolic engineering, our future research will be directed toward the intensification of the light-driven Baeyer–Villiger oxidation.

Here, photobioreactors using the principle of internal illumination present themselves as a highly promising solution in order to alleviate the cell density limitation of cyanobacterial biocatalysts.^35,36^

## Supporting Information Available

Complete experimental procedures and strain information (PDF)
