# Supplementary material for: Photobiocatalytic Oxyfunctionalization with High Reaction Rate using a Baeyer–Villiger Monooxygenase from Burkholderia xenovorans in Metabolically Engineered Cyanobacteria
Source: ACS Catal. 2021 Dec 10;12(1):66–72. doi: 10.1021/acscatal.1c04555 (PMC8751089; doi:10.1021/acscatal.1c04555)
Supplement: Supplementary file 1 — cs1c04555_si_001.pdf [file cs1c04555_si_001.pdf]

Supporting Information

Photobiocatalytic Oxyfunctionalization with High Reaction Rate using a Baeyer-Villiger Monooxygenase from *Burkholderia xenovorans* in Metabolically Engineered Cyanobacteria

Elif Erdem‡ [a, b], Lenny Malihan-Yap‡ [a], Leen Assil-Companioni[a, c], Hanna Grimm[a], Giovanni Davide Barone[a], Carole Serveau-Avesque[b], Agnes Amouric[b], Katia Duquesne[b], Véronique de Berardinis [d], Yagut Allahverdiyeva[e], Véronique Alphand*[b], Robert Kourist*[a, c]

[a] Institute of Molecular Biotechnology, Graz University of Technology, Petersgasse 14

8010 Graz, Austria

[b] Aix Marseille Univ, CNRS, Centrale Marseille, iSm2 UMR7313, 13397 Marseille, France

[c] ACIB GmbH, 8010 Graz, Austria

[d] Génomique métabolique, Genoscope, Institut François Jacob, CEA, CNRS, Univ Evry, Université Paris-Saclay, 91057 Evry, France

[e] Department of Biochemistry, Faculty of Science and Engineering, University of Turku, Turku 20014, Finland

‡authors contributed equally to this work

*corresponding authors: kourist@tugraz.at and v.alphand@univ-amu.fr

Table of Contents

Experimental Procedures 3
1. Reagents 3
2. Strains and plasmids used throughout the study 3
3. List of Primers used throughout the study 4
4. Correlation of Cell Dry Weight and Optical Density at 750 nm (OD_{750}) 4
5. Media for Cultivation and whole-cell biotransformations 4
6. Selection of BVMOs from High-Throughput screening 5
7. Construction of *Synechocystis* mutants 5
8. Cultivation and Maintenance of *Synechocystis* strains 6
9. Transformation of mutants and segregation check 7
10. Whole-cell Biotransformations 8
11. Sample analysis using Gas Chromatography (GC) 8
12. Statistical Analysis 9

Results and Discussion 10
13. Phylogenetic Tree of *Burkholderia xenovorans* 10
14. Purification of $BVMO_{Xeno}$ and $CHMO_{Acineto}$ 12
15. Kinetic analysis 14
16. Determination of degradation temperatures (T_m) of $BVMO_{Xeno}$ 14
17. Determination of *in vitro* activity of Syn::$P_{cpc}BVMO_{Xeno}$ and its ΔFlv1 mutant 15
18. Application of substituted cyclic ketones with Syn::$P_{cpc}BVMO_{Xeno}$ 16
19. Whole cell Biotransformations using Syn::$P_{cpc}CHMO_{Acineto}$ 16
20. Whole cell biotransformation of **1a** using $BVMO_{Xeno}$ with different promoters 17
21. Inhibition studies in Syn::$P_{cpc}BVMO_{Xeno}$ 18

References 19

Experimental Procedures

1. Reagents

All chemicals were purchased either from Sigma-Aldrich (Steinheim, Germany) or Carl-Roth GmbH (Karlsruhe, Germany) unless otherwise mentioned and were used without further purifications.

2. Strains and plasmids used throughout the study

Table S1. List of strains and plasmids used during the study.

Strain/Plasmid	Description	Reference
SynRekB_P_{cpc}BVMO$_{Xeno}$	Integrative plasmid with P_{cpc} promoter, BVMO from *Burkholderia xenovorans*	This work
SynRekB_P_{cpc}BVMO$_{Parvi}$	Integrative plasmid with P_{cpc} promoter, BVMO from *Parvibaculum lavamentivorans*	This work
SynRekB_P_{cpc}CHMO	Integrative plasmid with P_{cpc} promoter, CHMO from from *Acinetobacter* sp.	This work
Synechocystis sp. PCC 6803	Cyanobacteria utilised for whole-cell biotransformations; geographical origin from California (USA)	[1]
Synechocystis sp. PCC 6803 ΔFlv1	*Synechocystis sp.* PCC 6803 knockout (KO) strain lacking the Flv1 protein (provided by Prof. Yagut Allahverdiyeva)	[2]
Escherichia coli BL-21 (DE3)	F– *omp*T *hsd*SB(rB–, mB–) *gal dcm* (DE3) *rrnB3* Δ*lacZ4787* *hsdR514* Δ(*ara*BAD)	Novagen
pET22b_*bvmo*$_{Parvi}$	pET22b LIC derivative carrying *bvmo*$_{Parvi}$ gene with a N terminus 6-His tag (ampicillin resistance)	[3]
pET22b_*chmo*$_{Acineto}$	pET22b LIC derivative carrying *chmo*$_{Acineto}$ gene with a 6-His tag (ampicillin resistance)	This work
pET22b_*bvmo*$_{Xeno}$	pET22b LIC derivative carrying *bvmo*$_{Xeno}$ gene with a N-terminus 6-His tag (ampicillin resistance)	This work
ΔFlv1::P_{cpc}BVMO$_{Xeno}$	Transgenic *Synechocystis sp.* PCC 6803 strain harbouring the *bvmo*$_{Xeno}$ gene under the control of the P_{cpc} promoter in the genome locus *slr0168*. The strain was constructed using the ΔFlv1 background *via* homologous recombination using SynRekB_P_{cpc}BVMO$_{Xeno}$.	This work
ΔFlv1::P_{cpc}BVMO$_{Parvi}$	Transgenic *Synechocystis sp.* PCC 6803 strain harbouring the *bvmo*$_{Parvi}$ gene under the control of the P_{cpc} promoter in the genome locus *slr0168*. The strain was constructed using the ΔFlv1 background *via* homologous recombination using SynRekB_P_{cpc}*bvmo*$_{Parvi}$.	This work

3. List of Primers used throughout the study

Table S2. The primers used during PCR for Gibson assembly.

Amplification target	*Purpose*	*Primer sequence (5'→3')*
Plasmid	SynRekB Plasmid_FW	GGATCCGCGACCCATTTG
	SynRekB Plasmid_REV	GCAGATCTCGAGCTCGGTTTATTTTTC
Insert	$bvmo_{parvi}$_FW	AGCAAATGGGTCGCGGATCCATGAGTTCGGTACAATCC
	$bvmo_{parvi}$_REV	AAACCGAGCTCGAGATCTGCTCAATCCAAGACGAATCC
Plasmid	SynRekB PlasmidXeno_FW	GCAGATCTCGAGCTCGGTTTATTTTTCTA
	SynRekB PlasmidXeno_REV	GGATCCGCGACCCATTTGCTGT
Insert	$bvmo_{xeno}$_FW	CAAATGGGTCGCGGATCCATGACAACGAAGGGAAACGA
	$bvmo_{xeno}$_REV	TAAACCGAGCTCGAGATCTGCTCAGGCAGGCTCGACA

4. Correlation of Cell Dry Weight and Optical Density at 750 nm (OD_{750})

An OD_{750} of 10 corresponds to 2.4 g_{DCW} L^{-1} as previously determined for *Synechocystis* sp. PCC6803.[4,5]

5. Media for Cultivation and whole-cell biotransformations

For *E. coli*

Lysogeny Broth (LB, 1 L: Tryptone 10 g, Yeast Extract 5 g, NaCl 5 g) was utilized for 5 mL overnight cultures which were then transferred to Terrific Broth (TB, 1L: Peptone 12 g, Yeast extract 24 g, Glycerol 4 mL, KH_2PO_4 2.2 g, K_2HPO_4 9.4 g) media for protein production biotransformation. Liquid media were autoclaved at 121 °C for 21 minutes and kept at room temperature. Solid media were prepared in LB agar plates (15 g Agar L^{-1}). Ampicillin (Amp, 100 µg mL^{-1}) was utilized as selection marker.

For *Synechocystis*

Standard BG-11[4] was utilized for the cultivation of Syn::P_{cpc}BVMO$_{Xeno}$ and Syn::P_{cpc}BVMO$_{Parvi}$ as well as their ΔFlv1 variants. A modified medium for whole-cell biotransformations termed as BG-11/MOPS was utilized. As selection marker, Kanamycin

(Kan) was used at a final concentration of 50 μg mL^{-1}. Stock solutions (50 mg mL^{-1}) were prepared, sterile filtered (0.2 μm) and stored at -20 °C.

MOPS buffer (1 L, 0.4 M, 10x) was prepared by combining 400 mM of MOPS (3-(*N*-morpholino)propanesulfonic acid), 400 mM of sodium acetate and 10 mM of EDTA (Ethylenediaminetetraacetic acid) in nuclease-free distilled water. Prior usage, the solution's pH was adjusted to 7 using NaOH and autoclaved as described above.

BG-11/MOPS buffer (900 mL) was prepared by combining 90 mL of MOPS buffer (10X) with standard BG-11 (without HEPES and EDTA). The solution was autoclaved prior usage and has a resulting pH of 6.8-7.

6. Selection of BVMOs from High-Throughput screening

The sequences of 28 experimentally confirmed type I BVMOs were used for a blast-based screening against 1500 sequences from the public databases (Genoscope, CEA, Evry, France - Dr. V. de Bérardinis).[3,6] The gene candidates were chosen depending on their accessibility in the Genoscope strain collection. After clustering, 450 putative *bvmo* genes were cloned in *E. coli* BL21(DE3) then 370 candidate enzymes were produced and sixty active enzymes were identified, selected out of a high throughput screening (HTS) over twenty substrates. In this study, eleven enzymes from this screening were tested *via* whole cell experiments against cyclohexanone.

7. Construction of *Synechocystis* mutants

SynRekB_P$_{cpc}$_*chmo* and SynRekB_P$_{PsbA2}$_*chmo* plasmids were kindly provided by Prof. Dr. Robert Kourist (Graz University of Technology, Graz, Austria). BVMO$_{Parvi}$ (accession number: A7HU16) and BVMO$_{Xeno}$ (accession number: Q13I90) are coded by the genes *bvmo*$_{Parvi}$ and *bvmo*$_{Xeno}$, respectively. The genes were obtained from high throughput cloning and were available in a modified pET22b plasmid (Genescope).[3] The two *bvmo* genes were inserted into the SynRekB::P$_{cpc}$ and SynRekB::P$_{PsbA2}$ vectors resulting in SynRekB His-BVMO$_{Parvi}$ and SynRekB His-BVMO$_{Xeno}$ plasmids. All of the constructs contain a 6-His tag in the N-terminus. Cloning into the SynRekB plasmids was performed using the Gibson

Assembly®. Furthermore, primers were designed according to the protocol and NEB Gibson assembly user manual.

Cloning Process: Using Q5 High-Fidelity DNA Polymerase (New England Biolabs, NEB), the overlapping insert is amplified with the pET22b_*bvmo*$_{Parvi}$ (< 5 ng) as a template and the SynRekB_P$_{cpc}$ vector is linearized by inverse PCR. Digestions of the PCR products were then performed separately with *Dpn*I (NEB) for 60 min at 37 °C. The assembly reaction contained approximately 100 ng of vector and 50 ng of the insert (molar ratio vector: insert of 1: 2) and was incubated at 50 °C for 20 min following the manufacturer's protocol.

After the assembly reaction, the reaction mixture was transformed into NEB 5-alpha *E. coli* cells (NEB). The transformed cells were spread onto LB agar plates containing kanamycin (50 µg mL^{-1}). For recombinant clone screening, colony PCR was performed using Illustra PuReTaq Ready-To-Go PCR Beads (GE Healthcare Life Sciences). After overnight growth of a selected clone at 37 °C, the recombinant plasmid (SynRekB_P$_{cpc}$_*bvmo*$_{Parvi}$) was extracted using a plasmid extraction miniprep kit (QIAGEN). Correct insertion was confirmed by sequencing (GATC Biotech and Microsynth AG).

8. Cultivation and Maintenance of *Synechocystis* strains

Seed cultures (in BG-11 liquid media or on BG-11 agar plates) were maintained in a plant growth chamber (SWGC-1000, WISD) at 30 °C under atmospheric CO_2 conditions and 50% humidity. Continuous illumination was provided by fitted white fluorescent lamps with an intensity between 40 to 60 µE m^{-2} s^{-1}. Cells were cultured in 100 mL or 300 mL Erlenmeyer flasks with 50 mL and 100 mL of working volumes, respectively on rotary shakers at 140 rpm. Kanamycin (50 µg mL^{-1}) was supplemented for all strains containing an expression cassette for BVMO$_{Parvi}$ and BVMO$_{Xeno}$. For ΔFlv1 variants, strains were also supplemented with 10 µg mL^{-1} of chloramphenicol together with kanamycin (50 µg mL^{-1}).

For higher light cultivations, *Synechocystis* strains were cultivated in standard BG-11, supplemented with the appropriate antibiotics. The cultures were placed on a rotary shaker at 140 rpm and illuminated by a tunable LED lamp (CellDEG, Berlin, Germany) emitting red and blue light at an intensity of 150 µE m^{-2} s^{-1} as previously described.[5] *Synechocystis* cells harboring BVMO$_{Xeno}$ and BVMO$_{Parvi}$ were grown until an OD_{750}= 0.80-1.20 and OD_{750}= 1-2, respectively. On the other hand, ΔFlv1 variants were grown until an OD_{750} of 1.5-2.0.

9. Transformation of mutants and segregation check

Wild-type cells were cultivated and harvested at an optical density of OD_{750} = 0.5-1. The cells were then re-suspended in fresh BG-11 medium (500 µL). Plasmid DNA (5 µg) containing the vector was added to the cell suspension and were incubated at 30 °C for 6 h in darkness with shaking (140 rpm). Afterwards, the cells were transferred onto a sterile nitrocellulose membrane (GE Healthcare) and placed on a BG-11 agar plate without antibiotics. Following overnight incubation at low-intensity light conditions (30 µmol photons m^{-2} s^{-1}), the membrane was transferred to agar plates containing low concentrations of antibiotics (10-25 µg mL^{-1}) until colonies are noticeable. Colonies were then transferred gradually to BG-11 agar plates containing increasing concentration of kanamycin (50-100 µg mL^{-1}). For the ΔFlv1 mutants, chloramphenicol (10 µg mL^{-1}) was added in addition to kanamycin (50 µg mL^{-1}) as a selective marker. Figure S1 shows the segregation check for ΔFlv1::P_{cpc}BVMO$_{Parvi}$ and ΔFlv1::P_{cpc}BVMO$_{Xeno}$.

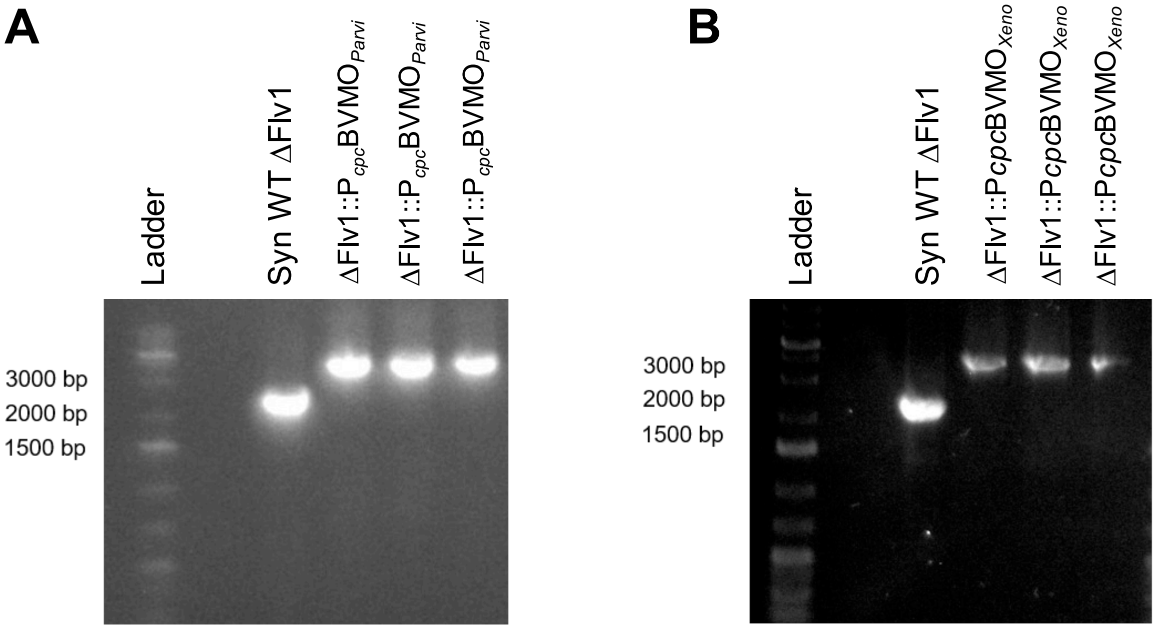

Figure S1. Segregation check of (A) ΔFlv1::P_{cpc}BVMO$_{Parvi}$ and (B) ΔFlv1::P_{cpc}BVMO$_{Xeno}$. Ladder = GeneRuler™ 1 kb DNA Ladder, Thermo Fischer Scientific.

10. Whole-cell Biotransformations

E. coli

Culture: 30 mL TB medium in baffled flasks were inoculated from overnight pre-cultures of the BVMO producing strains. The cells were grown at 37 °C and 160 rpm in the Innova42 incubator until the OD_{600} reached 2-3. Then they were induced by 1 mM of IPTG (final concentration) and incubated at 25 °C until the OD_{600} reached 7-8. Production of the target enzyme was checked by SDS Page analysis (see Figure S4).

Biotransformation: the flasks were transferred to a water bath shaker (25 °C) and 5 mM cyclohexanone was added. Samples were taken at different time points and extracted with ethyl acetate containing 0.5 g L^{-1} of decane as internal standard. The activity (ε-caprolactone formation rate) is determined over the first 90 min.

Synechocystis

After reaching the desired OD_{750} (see Section 8), Synechocystis strains producing BVMOs were concentrated by centrifugation (24 °C, 15 min, 3220 *g*) and the pellet was re-suspended in fresh BG-11/MOPS medium (pH 7) to an OD_{750} of 20-25. Biotransformation reactions were performed at 30 °C in 5 mL glass vials with a working volume of 1 mL under a constant light regime of 300 μE m^{-2} s^{-1} delivered by a tunable LED lamp emitting mostly blue and red lamp (CellDEG, Berlin, Germany). The reaction was initiated by combining BG-11/MOPS (590 μL), cell suspension (OD_{750} = 25, 400 μL) and substrate stock solution (1 M in ethyl acetate, 10 μL) in a glass vial. Aliquots of the reaction mixture were taken at different time intervals, quenched in liquid nitrogen and kept at -20 °C prior analysis.

11. Sample analysis using Gas Chromatography (GC)

Achiral compounds

Samples (100 μL) were extracted with dichloromethane (300 μL) containing 2 mM of *n*-decanol as internal standard. The suspension was mixed by inverting up and down for 1 min and the organic layer was dried using one spatula tip of anhydrous $MgSO_4$. After centrifugation (4 °C, 5 min, 13 000 *g*), the organic phase was separated and measured directly on achiral GC-FID using nitrogen as the carrier gas with a split ratio of 20 as previously described.[5] All

compounds stemming from the oxidation of cyclohexanone (**1a**) to its corresponding lactone (**1b**) and side-product (**1c**) was determined using GC equipped with a Flame Ionization Detector (FID, GC-2010 Plus, Shimadzu, Japan). The GC-FID system was outfitted with an achiral ZB-5 column. Separation of cyclohexanone, ε-caprolactone and cyclohexanol was performed at 60 °C (hold 5 min), 200 °C (hold 3 min, 10 °C min^{-1}) to 300 °C (hold 3 min, 25 °C min^{-1}). Retention times cyclohexanone: 7.7 min, ε-caprolactone: 13.0 min, cyclohexanol: 7.5 min, *n*-decanol: 15.0 min

Chiral compounds

Samples (150 µL) were extracted with EtOAc (300 µL) containing 5 mM of tetradecane as internal standard. After centrifugation (2 min, 13000*g*), the organic phase was separated and measured directly on chiral GC-FID. GC-FID analyses were conducted on a Shimadzu GC-2010 with hydrogen as the carrier gas and a split ratio of 20. Separation was achieved on a Chirasil-Dex (Varian capillary columns). Separation of 2-Phenylcyclohexanone and its corresponding lactones was performed at 100 °C to 160 °C (hold 10 min) with 2 °C min^{-1} temperature increase. Retention times 2-Phenylcyclohexanone (*S*): 9.2 min, 2-Phenylcyclohexanone (*R*): 9.4 min, tetradecane: 8.9 min, 7-phenyloxepan-2-one (*S*) 30.6 min, 7-phenyloxepan-2-one (*R*) 30.8 min.

12. Statistical Analysis

Statistical analysis was performed using GraphPad Prism version 8.0. Shapiro-Wilk's test was used to assess the normality of the data sets. Unpaired Welch's *t* test was then utilized for comparison of two data sets following a Gaussian distribution. A minimum of three independent (N=3) measurements or calculations were utilized for the statistical analyses (α = 0.05).

Results and Discussion

13. Phylogenetic Tree of *Burkholderia xenovorans*

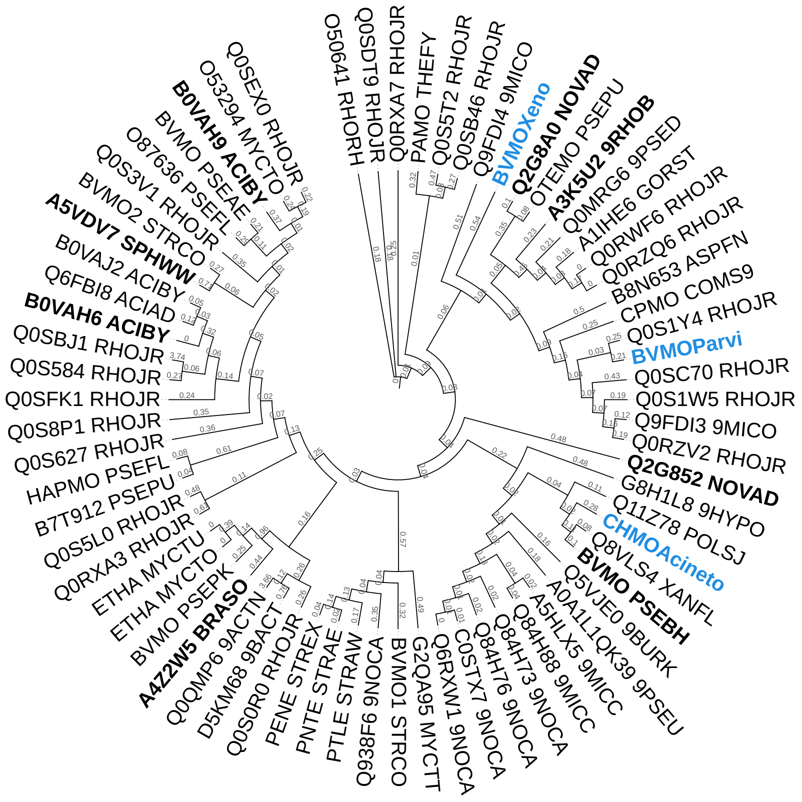

Figure S2. Phylogram showing seventy Type I BVMOs. The BVMOs screened in this study are shown in bold or blue.

The circular, rooted phylogenetic tree (Figure S4) was generated by using ngphylogeny.fr [7] The accession numbers of the selected enzymes are: A5HLX5_9MICC, B0VAJ2_ACIBY, B7T912_PSEPU, O53294_MYCTO, O87636_PSEFL,ETHA_MYCTO, ETHA_MYCTU, Q0MRG6_9PSED, PAMO_THEFY, Q5VJE0_9BURK,Q6FBI8_ACIAD, Q84H73_9NOCA, Q84H76_9NOCA, Q84H88_9MICC, BVMO_PSEPK, CPMO_COMS9, Q8VLS4_XANFL, Q938F6_9NOCA, HAPMO_PSEFL, Q9FDI3_9MICO, Q9FDI4_9MICO, CHMOAcineto (Q9R2F5) , A1IHE6_GORST, C0STX7_9NOCA, D5KM68_9BACT, PNTE_STRAE, PENE_STREX, Q0QMP6_9ACTN, Q0RWF6_RHOJR, Q0RXA3_RHOJR, Q0RXA7_RHOJR, Q0RZQ6_RHOJR, Q0RZV2_RHOJR, Q0S0R0_RHOJR, Q0S1W5_RHOJR, Q0S1Y4_RHOJR, Q0S3V1_RHOJR, Q0S584_RHOJR, Q0S5L0_RHOJR, Q0S5T2_RHOJR, Q0S627_RHOJR,

Q0S8P1_RHOJR, Q0SB46_RHOJR, Q0SBJ1_RHOJR, Q0SC70_RHOJR, Q0SDT9_RHOJR, Q0SEX0_RHOJR, Q0SFK1_RHOJR, Q6RXW1_9NOCA, PTLE_STRAW, BVMO_PSEAE, BVMO2_STRCO, BVMO1_STRCO, G8H1L8_9HYPO, Q11Z78_POLSJ, A5VDV7_SPHWW, Q2G852_NOVAD, Q2G8A0_NOVAD, B0VAH9_ACIBY, B0VAH6_ACIBY, A3K5U2_9RHOB, A4Z2W5_BRASO, B8N653_ASPFN, A0A1L1QK39_9PSEU, OTEMO_PSEPU, BVMOParvi (A7HU16), G2QA95_MYCTT, BVMOXeno (Q13I90, in this study), BVMO_PSEBH, O50641_RHORH.

The newly discovered enzyme (accession number Q13I90) from the bacteria *Burkholderia xenovorans* ($BVMO_{Xeno}$) has a percentage identity of 40% with $CHMO_{Acineto}$. The typical amino acid signature of Type I BVMO is present but the typical class B signature is slightly modified in a manner never described until now in the BVMO family.

Alignment of five different BVMOs, $CHMO_{Acineto}$, $BVMO_{Xeno}$, $BVMO_{OTEMO}$, $BVMO_{Parvi}$, and $BVMO_{PAMO}$, respectively, was performed using Clustal W (https://www.ebi.ac.uk/Tools/msa/clustalo/). Type I BVMO motif, [A/G]GxWxxxx[F/Y]P[G/M]-xxxD, is enclosed in rectangular boxes.

CLUSTAL O(1.2.4) multiple sequence alignment

```
CHMOAcineto     -----------MSQKMDFDAIVIGGGFGGLYAVKKLRDELELKVQAFDKATDVAGTWYWN    49
BVMOXeno        --MTTKGNDKNPAKSVDFEAIIVGAGFGGIRALHEMR-KLGLSVRLFEAGSDVGGTWYWN    57
OTEMO_PSEPU     --------MSNRAKSPALDAVVIGAGVTGIYQAFLIN-QAGMKVLGIEAGEDVGGTWYWN    51
BVMOParvi       MSSVQSSQTQKNDDAEVFDALIVGAGFNGIYQLHRLR-QEGFKVRLFEAGADMGGIWYWN    59
PAMO_THEFY      -MAGQTTVDSRRQPPEEVDVLVVGAGFSGLYALYRLR-ELGRSVHVIETAGDVGGVWYWN    58
                                 .:.:::*.*. *:     :. :   .*  :: . *:.* ****

CHMOAcineto     RYPGALTDTETHLYCYSWDKELLQSLEIKKKYVQGPDVRKYLQQVAEKHDLKKSYQFNTA    109
BVMOXeno        RYPGARTDSESWSYCFYFSRELMEEWNWPERMPSWQHVQNYMRYTVDRFGMRTDMQFDTR    117
OTEMO_PSEPU     RYPGCRLDTESYAYGYFALKGIIPEWEWSENFASQPEMLRYVNRAADAMDVRKHYRFNTR    111
BVMOParvi       CYPGARVDSHIPIYEFSI-EELWRDWNWTERFPAWDELRRYFHYVDKKLDLSRDIRFGMR    118
PAMO_THEFY      RYPGARCDIESIEYCYSFSEEVLQEWNWTERYASQPEILRYINFVADKFDLRSGITFHTT    118
                 ***.  * .   * :   . :  . :  :.     .: .*.. . .  .:     *

CHMOAcineto     VQSAHYNEADALWEVTTEYGDKYTARFLITALGLLSAPNLPNIKGINQFKGELHHTSRWP    169
BVMOXeno        VRSAHYNEEENHWTITTEQGESYTCTYFISAIGWFEVPTKPEFKGLDSFAGQWYLSCRWP    177
OTEMO_PSEPU     VTAARYVENDRLWEVTLDNEEVVTCRFLISATGPLSASRMPDIKGIDSFKGESFHSSRWP    171
BVMOParvi       VSAAEFDEARDQWVIRTTDGTVVRARFFILCTGFASKPYIPNYKGLESFAGESFHTGLWP    178
PAMO_THEFY      VTAAAFDEATNTWTVDTNHGDRIRARYLIMASGQLSVPQLPNFPGLKDFAGNLYHTGNWP    178
                * :* : *    * :         . ::* . *  .    *:  *:..* *: . :  **

CHMOAcineto     DD-------VSFEGKRVGVIGTGSTGVQVITAVAPLAKHLTVFQRSAQYSVPIGNDPLSE    222
BVMOXeno        EK------PVDFTGKRVGIVGAGSTAVQIFTTVAKTAKHVSLFQRTPNYVMPSRNYPLDE    231
OTEMO_PSEPU     TDAEGAPKGVDFTGKRVGVIGTGATGVQIIPIAAETAKELYVFQRTPNWCTPLGNSPMSK    231
BVMOParvi       QE------GASFTGKRVGVVGTGASGVQVVQEASKDAAHLTVFQRTPILALPMQQRKLDV    232
PAMO_THEFY      HE------PVDFSGQRVGVIGTGSSGIQVSPQIAKQAAELFVFQRTPHFAVPARNAPLDP    232
                 .       ..* *:***::*:*::.:*:    :  * .: :***:     *  :  :.

CHMOAcineto     EDVKKIKDNYDKIWDGVWNSALAFGLNESTVPAMSVSAEERKAVFEKAWQTGGGFRFMFE    282
BVMOXeno        ADQLSIKRDYDAIVAQIRKQVFAFPMKDSTLRYDDVNDEQRQAILDAGWEAG-GFRFLFE    290
OTEMO_PSEPU     EKMDSLRNRYPTILEYVKSTDTAFPYHRDPRKGTDVSESERDAFFEELYRQP-GYGIWLS    290
BVMOParvi       ETQQRMKADYPEIFRIRRETFGGFDILRDERSALEVPPEERCALYEKLWQKG-GFHYWIG    291
```

```
PAMO_THEFY       EFLADLKKRYAEFREESRNTPGGTHRYQGPKSALEVSDEELVETLERYWQEG-GPDI-LA     290
                      ::  *  :      .   .     .     .*  .:     :  :.   *    :

CHMOAcineto      TFGDIATNMEANIEAQNFIKGKIAEIVKDPAIAQKLMPQ----DLYAKRPLCDSGYYNTF     338
BVMOXeno         TFCDIFVDERSNAAASEYVRNKIRAIVKDPVVAEKLCPK---YPIALKRPPTCNFYYETF     347
OTEMO_PSEPU      GFRDLLLNKESNKFLADFVAKKIRQRVKDPVVAEKLIPK--DHPFGAKRVPMETNYYETY     348
BVMOParvi        GFSDILTNEEANRTMYDFWRDKTRARIKNPALADKLAPMEPPHPFGVKRPSLEQWYYEAF     351
PAMO_THEFY       AYRDILRDRDANERVAEFIRNKIRNTVRDPEVAERLVPK--GYPFGTKRLILEIDYYEMF     348
                  : *:  :  :*    ::   *    :::* :*::* *      :  **      **: :

CHMOAcineto      NRDNVRLEDVKANPIVEITENGVKLENGDFVELDMLICATGFDAVDGNYVRMDIQGKNGL     398
BVMOXeno         NRSNVSLVDISESPIDEIVPGGIRVGA-DHHELDIIIFAMGFDAVTGPLSNLDVRGRGGE     406
OTEMO_PSEPU      NRDNVHLVDIREAPIQEVTPEGIKTAD-AAYDLDVIIYATGFDAVTGSLDRIDIRGKDNV     407
BVMOParvi        NQDNVSLVDVREMPIVEIVPEGVLTSD-GLVELDMLVLATGFDAVTGGLTQIDIHGTGGI     410
PAMO_THEFY       NRDNVHLVDTLSAPIETITPRGVRTSE-REYELDSLVLATGFDALTGALFKIDIRGVGNV     407
                 *:.** * *    **  :.  *:        :** :: * ****: *   .:*::* ..

CHMOAcineto      AMKDYWKEGPSSYMGVTVNNYPNMFMVLGPNGP--FTNLPPSIESQVEWISDTIQYTVEN     456
BVMOXeno         TIKNKWQAGPRTSLGIGIDGFPNLFIVTGPQAP--FANVPPIVEAAVTWIGHAITHMREK     464
OTEMO_PSEPU      RLIDAWAEGPSTYLGLQARGFPNFFTLVGPHNGSTFCNVGVCGGLQAEWVLRMISYMKDN     467
BVMOParvi        TLKEKWTEGARTYLGFATSGFPNMLFLYGPQSPSGFCNGPTCAEMQGEWVVDCLKHMREN     470
PAMO_THEFY       ALKEKWAAGPRTYLGLSTAGFPNLFFIAGPGSPSALSNMLVSIEQHVEWVTDHIAYMFKN     467
                  : : *  *  : :*.   .:**:: : **     : *          *:   : :  .:

CHMOAcineto      NVESIEATKEAEEQWTQTCANIAEMTLFPK---AQSWIFGANIPG---KKNTVYFYLGGL     510
BVMOXeno         GYDRMEASPEAVESWAQLMQTMVNATLLRHAGTQRSWYMGANIPG---KPQTVLFYFGGA     521
OTEMO_PSEPU      GFTYSEPTQAAENRWTEEVYADFSRTLLAE---ANAWWVKTTTKPDGSVVRRTLVHVSGG     524
BVMOParvi        NKGRIEATAQAEEEWAQLLNSIAGMTLFPR---ADSWYMGANIPG---KPRQLLNF-PGV     523
PAMO_THEFY       GLTRSEAVLEKEDEWVEHVNEIADETLYPM---TASWYTGANVPG---KPRVFMLYVGGF     521
                 .    *      : *.:        **        :*   :.         .    .  *

CHMOAcineto      KEYRSALANCKNHAYEGFDIQLQRSDIKQPANA 543
BVMOXeno         EAYFNDIERVAANGFEGFNFSRTGETVEPA--- 551
OTEMO_PSEPU      PEYRKRCEQVAYNNYNGFELA------------ 545
BVMOParvi        PIYMDQCNTAAAKDYEGFVLD------------ 544
PAMO_THEFY       HRYRQICDEVAAKGYEGFVLT------------ 542
                   * .       : ::** :
```

Figure S3. Clustal alignment of $BVMO_{Xeno}$ with four other BVMOs. Type I BVMO motif is shown in yellow.

14. Purification of $BVMO_{Xeno}$ and $CHMO_{Acineto}$

For purification of the 6xHis-tagged $BVMO_{Xeno}$ and $CHMO_{Acineto}$, the cells were grown at 37˚C and 160 rpm until OD_{600}= 0.8. After induction with 1 mM IPTG (final), the production of $BVMO_{Xeno}$ and $CHMO_{Acineto}$ were performed overnight at 18 ˚C and 15 ˚C, respectively. Protein purification was performed under native conditions according to Protino®-Macherey-Nagel Ni-NTA purification protocol. All buffers were prepared accordingly and stored at 4 ˚C. Purifications were performed at 4 ˚C and the enzymes were kept on ice throughout the whole process. After Ni-NTA affinity purification, imidazole was removed from the purified enzymes by utilizing desalting columns (GE healthcare) using Tris-HCl buffer, pH 8.0. The samples were then loaded to 10% SDS gel and were ran at 100 V. The images were taken following the Coomassie staining/destaining steps.

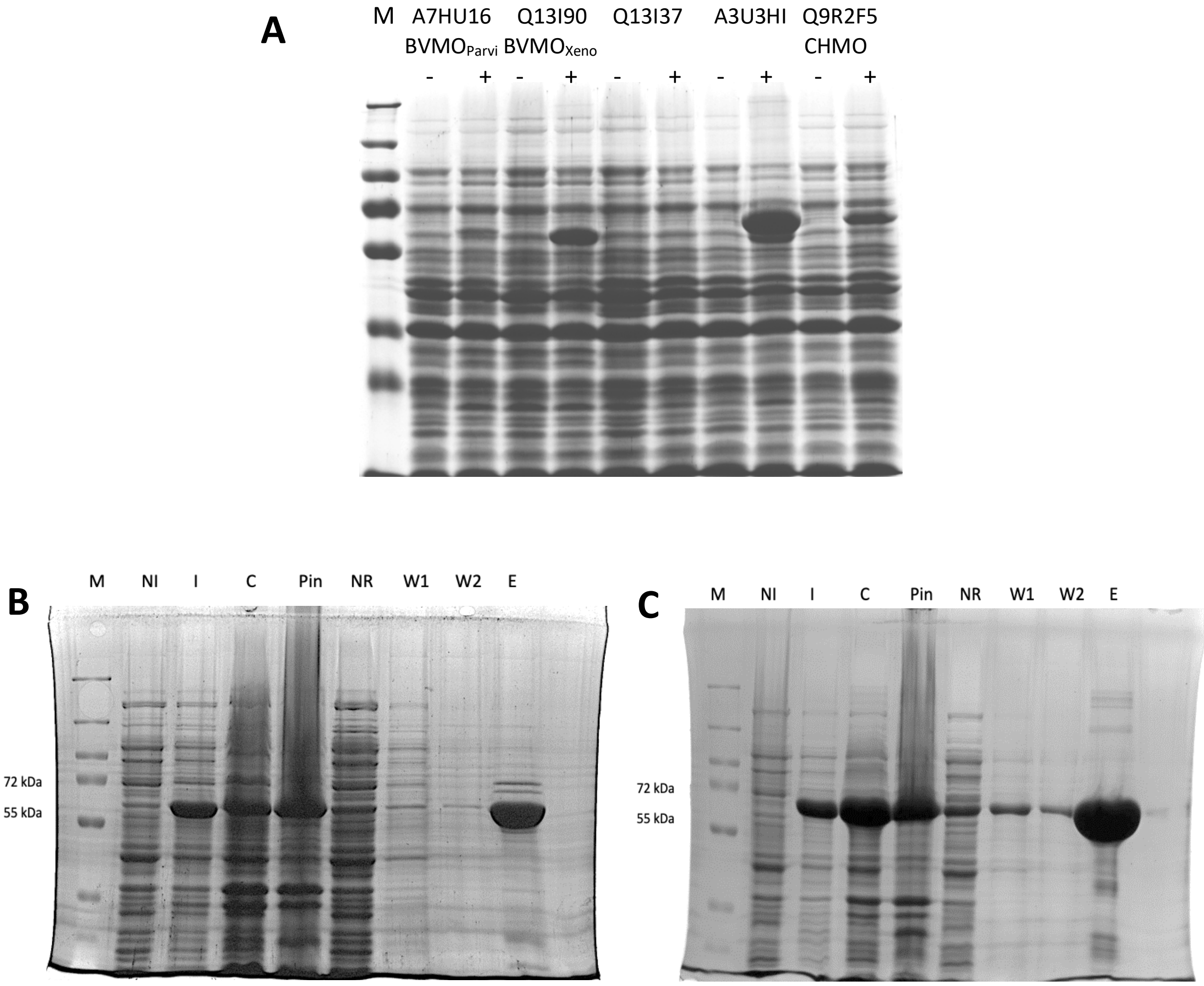

Figure S4. SDS-Page analyses: (A) Producing BVMO *E. coli* before whole cell biotransformations. Samples taken before (-) and after (+) induction., (B) Ni-NTA purified 6xHis tagged BVMO$_{Xeno}$ and (C) Ni-NTA Purified 6xHis tagged CHMO$_{Acineto}$ enzyme fractions from Ni-NTA column purification (M: Marker, NI: Non induced, I: Induced, C: charged, Pin: Pellet, NR: Non retained, W1-2: washed, E: Eluted proteins). The respective size of BVMO$_{Xeno}$ and CHMO$_{Acineto}$ is 62.3 kDa and 60.9 kDa.

15. Kinetic analysis

Kinetic analyses were performed by monitoring the NADPH consumption in 96 well plates using purified enzymes. A final concentration of 1 mM cyclohexanone, 0.25 g L^{-1} of NADPH, 400 nM of FAD were mixed to final concentration of 33 mg L^{-1} of purified enzyme in 50 mM Tris-HCl (pH 8). Absorption measurements were performed under 340 nm using microplate reader at 25 °C.

For the kinetic parameter determination, a concentration ranging from 1 μm to 10 mM of cyclohexanone was utilized. For the substrate scope, 1 mM final concentration of each substrate was used from 1M stock solution dissolved in ethanol. The results are reported in Table 1.

16. Determination of degradation temperatures (T_m) of BVMO$_{Xeno}$

The apparent melting point (T_m) of both purified enzymes were determined by an adapted ThermoFAD method.[8] The purified enzymes (40 µM stock solution) were transferred to a qPCR plate (Multiwell Plate 96 white, Roche) with sealing foil and were resuspended into various 50 mM buffers (see Table S3) to a final concentration of 25 µM. The sealed plate was incubated in a qPCR instrument (Light Cycler 480; Roche) with a melting curve program (1 min at 20°C followed by a ramp from 20 to 95 °C with a continuous increase rate of 1°C min^{-1}). Fluorescence was monitored (45 measures per degree) using a predefined filter (excitation 465 nm, emission 510 nm). All experiments were carried out in duplicates.

Table S3. Degradation temperatures of BVMO$_{Xeno}$ and CHMO$_{Acineto}$ at different buffers.

Enzymes	HEPES pH 7.5	Na Phosphate pH 7.5	K Phosphate pH 8	Tris pH 8	Average
BVMO$_{Xeno}$	38.4 ± 0.4	37.6 ± 0.1	38.6 ±0.2	37.4 ± 0.0	38.0 ± 0.6
CHMO$_{Acineto}$	34.2 ± 0.1	35.7 ± 0.1	35.8 ±0.1	33.8 ± 0.2	34.9 ± 1.0

17. Determination of *in vitro* activity of Syn::P_{cpc}BVMO$_{Xeno}$ and its ΔFlv1 mutant

Cell free extracts (CFE) were obtained by centrifugation (3 min, 4°C, 16 000*g*) of 1 mL of cells (OD_{750} = 20). The resulting pellet was washed with Phosphate Buffer Saline (PBS, 2 mL, pH = 7.4). After centrifugation, the pellet was re-suspended in PBS (200 µL) following the addition of the proteinase inhibitor, aminocaproic acid (1 mM) and three spatula of glass beads (φ = 0.25 mm). Cells were disrupted by vortexing at maximum speed for 30 s (4 times) with cooling breaks on ice (2 min) in between. After centrifugation, PBS (100 µL) was added to the resulting blue colored lysate. The protein content of the CFE was determined using the BCA Assay (ThermoFisher Scientific) using Bovine Serum Albumin (BSA, 25-2000 µg mL^{-1}) as standard.

Potassium buffer (20 mM, pH = 6.5) was degassed for 10-15 min by sparging with nitrogen. A solution containing **1a** (1 mM), NADPH (125 µM) in potassium buffer was prepared and placed in a glass cuvette with cover. The reaction was started with the addition of the CFE (100 µL) and the absorption was monitored at 340 nm for 15min. Background reactions were subtracted by performing the reaction without the substrate. The enzyme's in vitro activity was calculated using the slope and the extinction coefficient for NADPH (ε = 0.0045 L $µmol^{-1}$ cm^{-1}) normalized to the protein content determined from the BCA Assay. Figure S6 show a representative diagram from the in vitro assays.

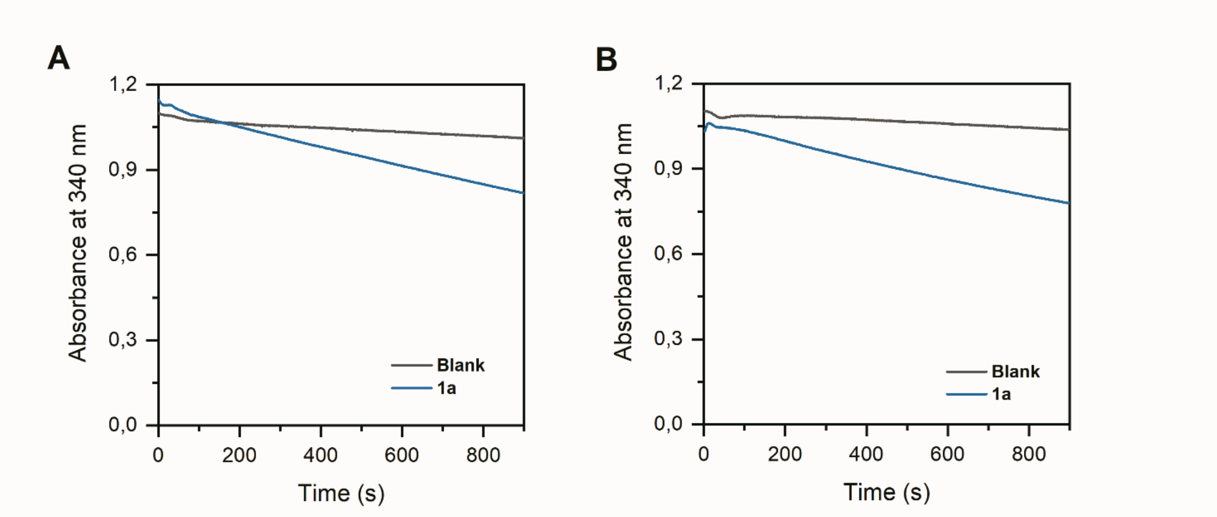

Figure S5. Time profile of NADPH absorbance in the presence of (A) Syn::P_{cpc}BVMO$_{Xeno}$ and (B) ΔFlv1::P_{cpc}BVMO$_{Xeno}$ during in vitro determination of the enzyme activity.

18. Application of substituted cyclic ketones with Syn::P$_{cpc}$BVMO$_{Xeno}$

Biotransformation with 2-Phenylcyclohexanone (**2a**) is performed with Syn::P$_{cpc}$BVMO$_{Xeno}$, resulting in full conversion of both ketones and E>200. The ee values were calculated from GC-FID analysis. Figure S7 shows the whole-cell biotransformation of 2-Phenylcyclohexanone using Syn::P$_{cpc}$BVMO$_{Xeno}$.

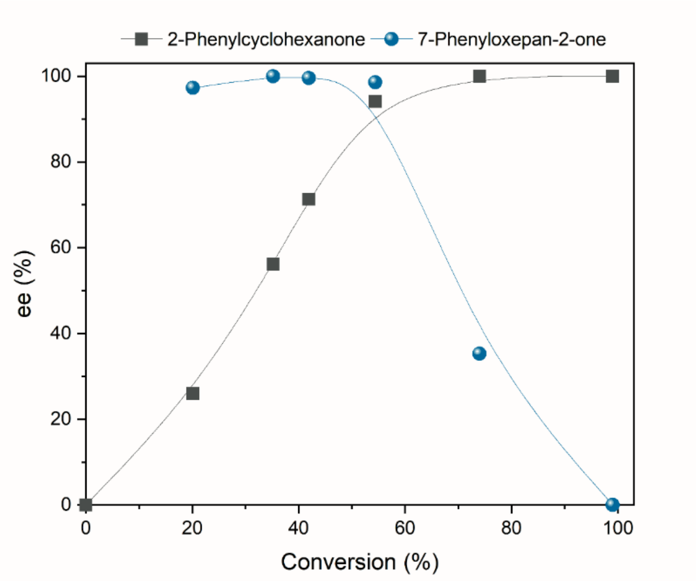

Figure S6. Enantiomeric excess from whole-cell biotransformations of **2a** (initial concentration of 10 mM) mediated by Syn::P$_{cpc}$BVMO$_{Xeno}$

19. Whole cell Biotransformations using Syn::P$_{cpc}$CHMO$_{Acineto}$

To be able to compare the specific activities with the same promoter, experiments were carried out using Syn::P$_{cpc}$CHMO$_{Acineto}$. Figure S8 shows the (A) Time course of product formation using Syn::P$_{cpc}$CHMO$_{Acineto}$ and (B) Specific activities comparison for Syn:: P$_{cpc}$CHMO$_{Acineto}$, Syn::P$_{cpc}$BVMO$_{Parvi}$ and Syn::P$_{cpc}$BVMO$_{Xeno}$. Specific activities were calculated based on ε-caprolactone formation.

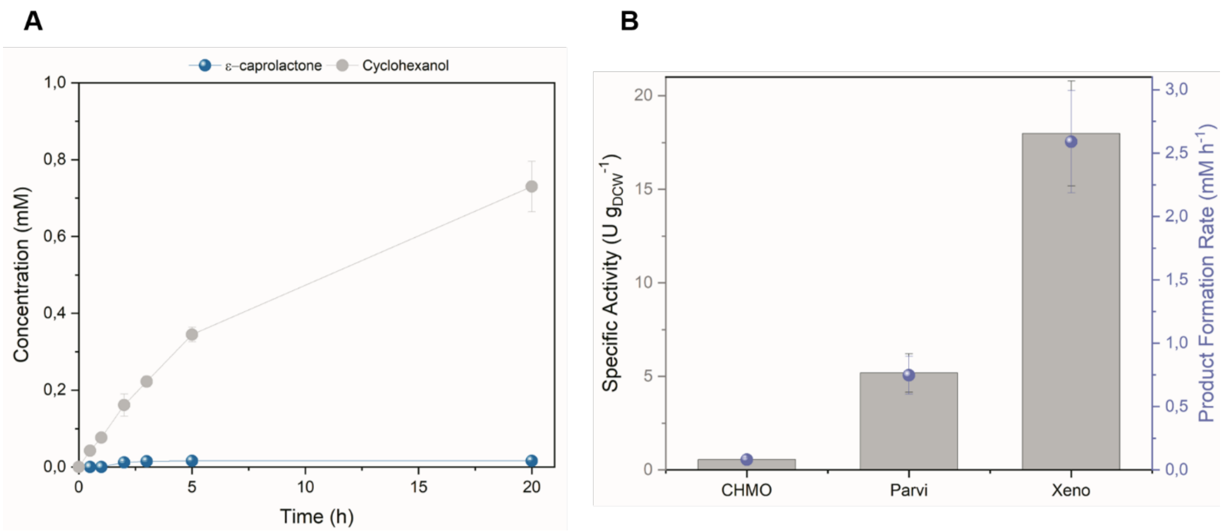

Figure S7. (A) Product formation during whole cell biotransformation of **1a** mediated by Syn::P$_{cpc}$CHMO$_{Acineto}$. Cyclohexanol **1c** (grey) and caprolactone **1b** (blue) concentration vs time. (B) Specific activity (bars) and **1b** formation rate (blue balls) with three *Synechocytis* strains producing BVMOs under P$_{cpc}$ control. *Reaction conditions*: V=1 mL, T= 30 °C, 300 µE m^{-2} s^{-1}, 160 rpm, N = 2-3, CDW =2.4 g L^{-1}.

20. Whole cell biotransformation of 1a using BVMO$_{Xeno}$ with different promoters

Synechocystis cells harboring BVMO$_{Xeno}$ controlled by the P$_{psbA2}$ promoter were cultivated similarly and utilized in whole-cell biotransformations of **1a**. Figure S8 show the specific activity comparison and time course of **1b** formation using Syn::P$_{cpc}$BVMO$_{Xeno}$ and Syn::P$_{psbA2}$BVMO$_{Xeno}$.

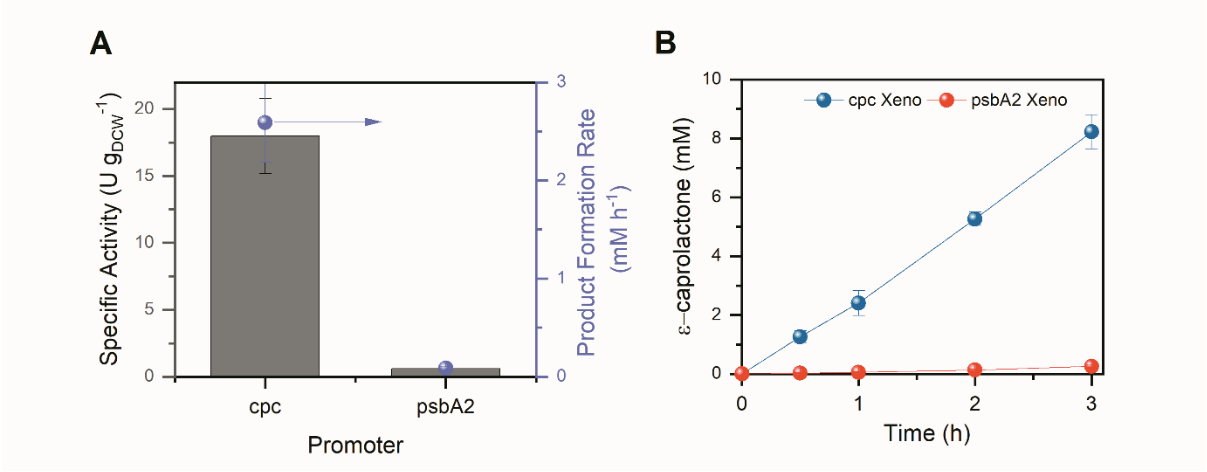

Figure S8. (A) Specific activities comparison between P_{cpc} and P_{psbA2} promoter and (B) ε-caprolactone formation during whole cell biotransformation of **1a** mediated by Syn::P$_{cpc}$BVMO$_{Xeno}$ and Syn::P$_{psbA2}$BVMO$_{Xeno}$ (B). *Reaction conditions*: V=1 mL, T= 30 °C, 300 µE m^{-2} s^{-1}, 160 rpm, N = 3, CDW = 2.4 g L^{-1}.

21. Inhibition studies in Syn::P$_{cpc}$BVMO$_{Xeno}$

To be able to study the extent of product inhibition, we ran whole cell biotransformations in Syn::P$_{cpc}$BVMO$_{Xeno}$ containing different concentrations of the product from the beginning of the reaction, ε-caprolactone (**1b**). The activity was calculated relatively to reactions without **1b**.

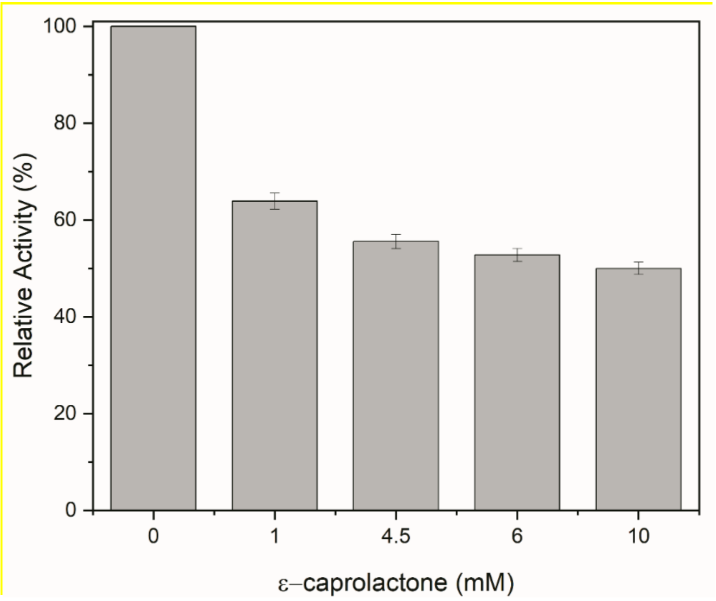

Figure S9. Effect of ε-caprolactone concentration during whole cell biotransformation of **1a** in Syn::P$_{cpc}$BVMO$_{Xeno}$. Reaction conditions: V=1 mL, T= 30 °C, 300 µE m^{-2} s^{-1}, 160 rpm, N = 3, CDW= 2.4 g L^{-1}, initial concentration of 10 mM **1a**.

References

(1) Stanier, R. Y.; Kunisawa, R.; Mandel, M.; Cohen-Bazire, G. Purification and Properties of Unicellular Blue-Green Algae (Order *Chroococcales*). *Bacteriological reviews* **1971**, *35*, 171–205. DOI:10.1128/mmbr.35.2.171-205.1971.

(2) Mustila, H.; Paananen, P.; Battchikova, N.; Santana-Sánchez, A.; Muth-Pawlak, D.; Hagemann, M.; Aro, E. M.; Allahverdiyeva, Y. The Flavodiiron Protein Flv3 Functions as a Homo-Oligomer during Stress Acclimation and Is Distinct from the Flv1/Flv3 Hetero-Oligomer Specific to the O_2 Photoreduction Pathway. *Plant and Cell Physiology* **2016**, *57*, 1468–1483. DOI:10.1093/pcp/pcw047.

(3) Reignier, T.; de Berardinis, V.; Petit, J.-L.; Mariage, A.; Hamze, K.; Duquesne, K.; Alphand, V. Broadening the Scope of Baeyer–Villiger Monooxygenase Activities toward α, β-Unsaturated Ketones: A Promising Route to Chiral Enol-Lactones and Ene-Lactones. *Chemical Communications* **2014**, *50*, 7793–7796.

(4) Büchsenschütz, H. C.; Vidimce-Risteski, V.; Eggbauer, B.; Schmidt, S.; Winkler, C. K.; Schrittwieser, J. H.; Kroutil, W.; Kourist, R. Stereoselective Biotransformations of Cyclic Imines in Recombinant Cells of *Synechocystis* sp. PCC 6803. *ChemCatChem* **2020**, *12*, 726–730. DOI:10.1002/cctc.201901592.

(5) Assil-Companioni, L.; Büchsenschütz, H. C.; Solymosi, D.; Dyczmons-Nowaczyk, N. G.; Bauer, K. K. F.; Wallner, S.; MacHeroux, P.; Allahverdiyeva, Y.; Nowaczyk, M. M.; Kourist, R. Engineering of NADPH Supply Boosts Photosynthesis-Driven Biotransformations. *ACS Catalysis* **2020**, *10*, 11864–11877. DOI:10.1021/acscatal.0c02601.

(6) Rebehmed, J.; Alphand, V.; De Berardinis, V.; De Brevern, A. G. Evolution Study of the Baeyer-Villiger Monooxygenases Enzyme Family: Functional Importance of the Highly Conserved Residues. *Biochimie* **2013**, *95*, 1394–1402. DOI:10.1016/j.biochi.2013.03.005.

(7) Lemoine, F.; Correia, D.; Lefort, V.; Doppelt-Azeroual, O.; Mareuil, F.; Cohen-Boulakia, S.; Gascuel, O. NGPhylogeny.Fr: New Generation Phylogenetic Services for Non-Specialists. *Nucleic Acids Research* **2019**, *47*, W260–W265. DOI:10.1093/nar/gkz303.

(8) Forneris, F.; Orru, R.; Bonivento, D.; Chiarelli, L. R.; Mattevi, A. ThermoFAD, a Thermofluor-adapted flavin ad hoc detection system for protein folding and ligand binding *FEBS J.* **2009**, *276*, 2833-2840. DOI:10.1111/j.1742-4658.2009.07006.x.
